# Traditional Chinese medicine Bu-Shen-Jian-Pi-Fang attenuates glycolysis and immune escape in clear cell renal cell carcinoma: results based on network pharmacology

**DOI:** 10.1042/BSR20204421

**Published:** 2021-06-10

**Authors:** Jinzhou Zheng, Wenhao Xu, Wangrui Liu, Haijia Tang, Jingen Lu, Kui Yu, Xiaoyun Song, Feng Li, Yu Wang, Rui Wang, Lili Chen, Hailiang Zhang, Yunhua Qiu, Gaomeng Wei, Xiqiu Zhou, Jianfeng Yang

**Affiliations:** 1 Department of Surgery, PuDong branch of Longhua Hospital, Shanghai University of Traditional Chinese Medicine, 1000 Shangnan road, Shanghai 200126, China; 2 Department of Urology, Fudan University Shanghai Cancer Center, Shanghai 200030, China; 3 Department of Urology, Affiliated Hospital of Youjiang Medical University for Nationalities, Guangxi 533000, China; 4 Department of Integrated Medicine, Nanjing University of Chinese Medicine, Nanjing 210000, China

**Keywords:** bioinformatics, Bu-Shen-Jian-Pi-Fang, clear cell renal cell carcinoma, glycolysis, network pharmacology, traditional Chinese medicine

## Abstract

Clear cell renal cell carcinoma (ccRCC) is the most common malignant type of kidney cancer. The present study aims to explore the underlying mechanism and potential targets of the traditional Chinese medicine Bu-Shen-Jian-Pi-Fang (BSJPF) in the treatment of ccRCC based on network pharmacology. After obtaining the complete composition information for BSJPF from the Traditional Chinese Medicine Systems Pharmacology Database and Analysis Platform, we analyzed its chemical composition and molecular targets and then established a pharmacological interaction network. Twenty-four significantly differentially expressed genes and nine pathways mainly related to tumor proliferation were identified and screened. Functional enrichment analysis indicated that the potential targets might be significantly involved in glycolysis and the HIF-1 signaling pathway. To further confirm the effect of BSJPF on ccRCC cell proliferation, a BALB/c xenograft mouse model was constructed. Potential targets involved in regulating glycolysis and the tumor immune microenvironment were evaluated using RT-qPCR. VEGF-A expression levels were markedly decreased, and heparin binding-EGF expression was increased in the BSJPF group. BSJPF also inhibited tumor proliferation by enhancing GLUT1- and LDHA-related glycolysis and the expression of the immune checkpoint molecules PD-L1 and CTLA-4, thereby altering the immune-rejection status of the tumor microenvironment. In summary, the present study demonstrated that the mechanism of BSJPF involves multiple targets and signaling pathways related to tumorigenesis and glycolysis metabolism in ccRCC. Our research provides a novel theoretical basis for the treatment of tumors with traditional Chinese medicine and new strategies for immunotherapy in ccRCC patients.

## Introduction

With the growth of the economy and improvements in living standards, the prevalence and mortality rates of kidney cancer are continuously increasing. Currently, kidney cancer accounts for ∼4% of all cancers worldwide. In recent years, there have been approximately 400,000 new kidney cancer cases and 180,000 related deaths per year [1]. The most common and deadliest form of kidney cancer is renal cell carcinoma (RCC) [2,3]. Clear cell renal cell carcinoma (ccRCC) accounts for approximately 85% of RCC cases. The metastasis of ccRCC is common, and about one-third of ccRCC patients have metastatic disease at the time of diagnosis [4]. At present, the first-line treatment for advanced ccRCC is mainly based on tyrosine kinase inhibitors targeting the vascular endothelial growth factor receptor [5]. Although anti-angiogenic drugs inhibit tumor proliferation to a certain extent and significantly prolong the survival of low-risk ccRCC patients, the use of these drugs is associated with adverse side effects. Furthermore, the objective response rate to this treatment is less than 30%, and the median survival time is less than 12 months [6–8]. In addition, even patients who achieve an initially effective treatment response will eventually experience disease progression and show a limited response to subsequent therapy [9]. Therefore, developing novel treatment strategies and potential targets for the treatment of ccRCC patients is urgently required.

Recently, traditional Chinese medicine prescriptions have been widely used to treat intractable diseases, such as tumors, diabetes, arthritis and others [10]. Bu-Shen-Jian-Pi-Fang (BSJPF) is a long-standing traditional Chinese medicine. Its main ingredients are Angelica, Salvia, Poria, Wolfberry, Pueraria Root, *Coptis chinensis, Ligustrum, Curcuma* and Coix Seed. Previous studies have shown that some components in BSJPF play a role in inhibiting tumor growth [10–12]. Interestingly, classic Chinese herbal formulas have been shown to have fewer side effects during treatment and be more cost-effective. Previous studies have found that the activation of immune cells and reprogramming of inflammatory responses associated with metabolism contribute to their effects [11–13].

To identify novel ccRCC treatment strategies, we investigated whether traditional Chinese medicine could inhibit the invasion and growth of ccRCC. To this end, we analyzed the key molecular targets and signaling pathways of BSJPF to clarify the mechanism underlying its specific biological effects and guide the clinical treatment of ccRCC. The present study aimed to explore the multi-component, multi-target and multi-pathway interactions and regulatory networks by which BSJPF inhibits ccRCC cell growth, induces cell cycle arrest and increases apoptosis based on network pharmacological analysis techniques. We hypothesized that an in-depth investigation of the underlying mechanism and potential targets of BSJPF could provide valuable information for the development of new effective drugs and strategies for the clinical treatment of ccRCC.

## Materials and methods

### Data collection and processing

The composition and molecular target data of BSJPF were obtained from the Traditional Chinese Medicine Systems Pharmacology Database and Analysis Platform (TCMSP) (http://tcmspw.com/tcmsp.php) [14], and the US Food and Drug Administration was used to compare the chemical structure. Validation data, including RNA-seq data of ccRCC tumor samples, were from the GEO database of the National Center for Biotechnology Information (https://www.ncbi.nlm.nih.gov/geo). Gene enrichment analysis data were obtained from the Bioinformatics Annotation Database (DAVID, https://david.ncifcrf.gov).

### Screening and identification for differential expressed genes (DEGs)

We compared the expression of genes in ccRCC cells and datasets with normal kidney tissue expression. In addition, we only selected datasets containing more than 20 samples. We screened the titles and abstracts of these datasets. Finally, only 44 datasets in GSE105261 were selected for further study. The GSE105261 dataset based on the GPL10558 platform was downloaded from the National Biotechnology Information Center and included 9 normal kidney tissues and 35 tumor samples.

### Protein target-ccRCC network construction

Cytoscape software (version 3.7.2) [15] was used to generate the protein target network (PPI) of the BSJPF target genes in ccRCC. The PPI was used to analyze the drug targets in TCMSP and the gene targets in ccRCC. We collected the data from six PPI databases for analysis, including the interaction protein database, biological universal interaction dataset knowledge base, human protein reference database, IntAct molecular interaction database, biomolecular interaction network database and molecular interaction function database. We built and plotted two PPI networks using Cytoscape software. After merging these two networks into candidate networks, we used topology analysis to gradually filter out the central network.

### Network topology analysis

Using the Cytoscape plug-in CytoNCA [16], we performed a topology analysis of the network. The filtering conditions were as follows: degree center (DC) > 61, topological intermediateness (BC) > 100, compactness center (CC) > 0.60, feature vector center (EC)> 0.02, local average connectivity (LAC) > 10 and network Center (NC) > 10 [17–19]. The definition and calculation equations of all parameters reflect the importance of the node. The importance of the node in the network was identified as the quantitative value. Through the above methods, we selected the central network.

### Construction of PPI network

We defined the tightly connected regions of molecular complexes in the obtained PPI network as topology modules with network characteristics [20,21]. The aggregation of similar and related nodes in the same pharmacological group was defined as a pharmacological module; the network that disrupts cell function or causes ccRCC proliferation was defined as a pathogenic module. In the topology analysis, the pharmacology module and pathogen module were in the same network. Using the Cytoscape plug-in MCODE to analyze the corresponding network, the final core PPI network cluster was obtained [21].

### Gene ontology (GO) and pathway enrichment analysis

We performed three biological analyses of the screened genes by GO analysis [22], including biological process (BP), cell composition (CC) and molecular function (MF). The annotation database was used to analyze the signaling pathways of the Kyoto Encyclopedia of Genes and Genomes (KEGG). We analyzed them using DAVID. A *P* value less than 0.05 was considered statistically significant. The ggplot2 plug-in package of R software (version 3.6.2) was used to visualize the above data.

### Survival analysis

A total of 530 ccRCC patients from the TCGA database with available mRNA sequence and survival outcome data were included. Kaplan–Meier Plotter (http://kmplot.com/analysis/) was utilized to perform survival analysis to identify the prognostic values of hub genes for BSJPF in ccRCC patients. Each survival analysis was implemented, all possible cutoff values between the lower and upper quartiles are computed, and the best performing threshold is used as a best cutoff in this study.

### Xenograft mouse model

For *in vivo* analysis, 6-week-old male nude mice were anesthetized, and the human ccRCC cell line RENCA (1 × 10^6^) premixed with Matrigel at a ratio of 1:2 was subcutaneously injected into the right rear back region. When tumors reached 120 mm^3^, mice were randomized into two groups (*n*=5 each), treated with a decoction of BSJPF or normal feeding and monitored for tumor size and volume weekly. Then, BALB/c mice were anesthetized with 2,2,2-Tribromoethanol (TEB, 200 mg/kg, No. S4508, Selleck, China) through intraperitoneal injection into the lower-right quadrant of the abdomen. The onset of anesthesia (the time to death by spinal dislocation) was defined as the time between the disappearance of the conditioned reflex and the recovery. After 6 weeks, the mice were killed using spondylolisthesis. All animal experiment procedures were carried out in the Medical Institution of Fudan University Shanghai Cancer Center (Shanghai, China) and approved by the Animal Care and Use Committee (No. 2008222-xp49).

### RT-qPCR assays

Total RNA was isolated after 3 days of culture using TRIzol reagent (Invitrogen, Carlsbad, CA, U.S.A.) in accordance with the manufacturer’s instructions. Potential targets involved in regulating glycolysis and the tumor immune microenvironment were detected. Significant hub genes and RT-qPCR reactions were carried out as previously described [23]. The mRNA expression level was normalized to beta-actin, and each reaction was performed in triplicate in accordance with the manufacturer's instructions. All primer sequences are listed in Supplementary Table S1.

### Statistical analysis

In the present study, R (Version 3.3.2), RStudio (Version 1.2) and GraphPad Prism (Version 8.0) were used to perform most data analyses. The Kaplan–Meier method with the 95%CI and log-rank test were used in separate survival curves. A two-tailed Student’s *t*-test or one-way ANOVA was used to measure differences between groups. All tests were two-sided, and *P* values less than 0.05 were considered significant.

## Results

**Figure 1:**
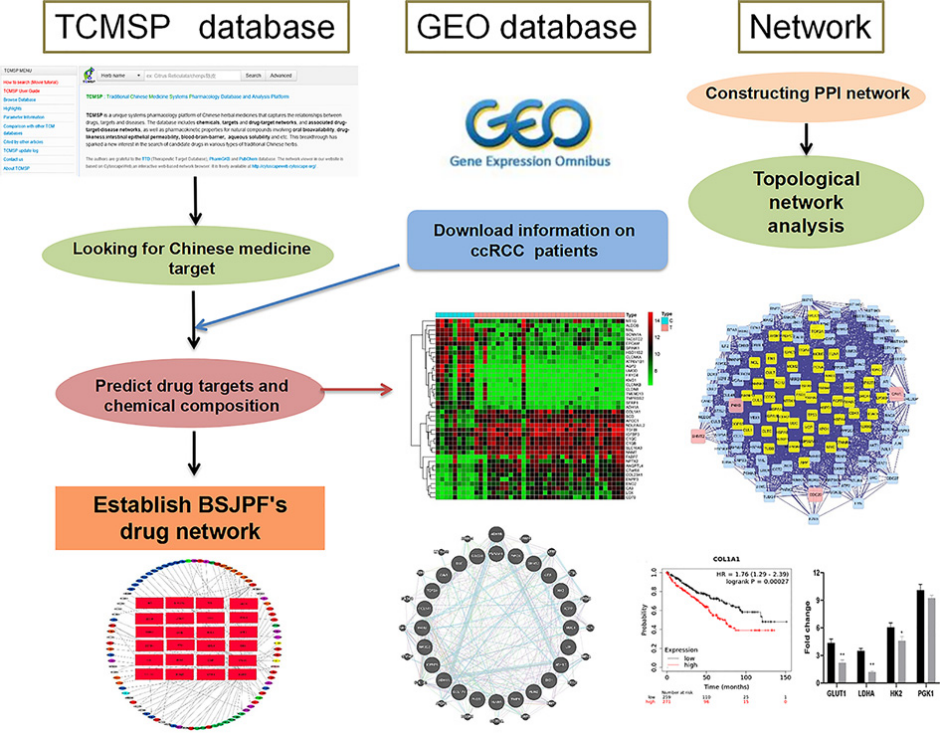
Flowchart of the systematic profiling of components and potential targets of BSJPF using Traditional Chinese Medicine Systems Pharmacology Database and Analysis Platform and TCGA databases in clear cell renal cell carcinoma in the present study

Our research was divided into three sequential stages, as illustrated in Figure 1. First, we downloaded the components of BSJPF in TCMIP and analyzed these target components to establish a drug network. Additionally, the data of ccRCC patients were downloaded from the GEO database, and the DEGs were screened to construct a tumor network. Next, we merged the two networks and performed topology analysis to identify the core network. We identified a total of 46 nodes and 583 edges. Finally, we conducted GO and KEGG analyses of these targets to investigate the mechanism underlying the effects of BSJPF in ccRCC patients.

### Identification of ccRCC differential genes

**Figure 2:**
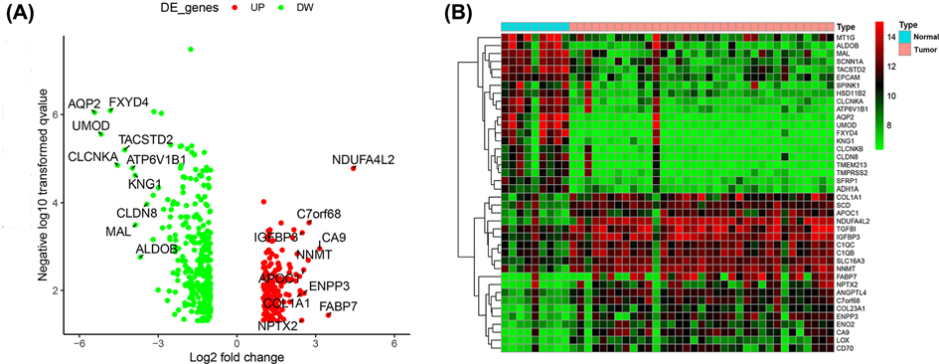
Identification of the difference expressed genes (**A**) Heat map showing expression levels differentially expressed genes in tumor and normal tissues. (**B**) Black dots indicate genes that are not differentially expressed between 9 normal kidney tissue samples and 35 tumor samples, and red and green dots indicate genes that are up- and down-regulated in tumor samples in the volcano map.

We selected GSE105261 downloaded from GEO as a sample, which contained 9 normal tissues and 35 tumor tissues. To identify DEGs, we performed differential expression analysis on the two sets of data obtained. Based on this analysis, we found that 455 genes in ccRCC were significantly differentially expressed compared with normal kidney tissue samples, including 165 up-regulated genes and 289 down-regulated genes. Tables 1 and 2 list the top 10 up-regulated genes and down-regulated genes, respectively. According to the fold change (FC), *NDUFA4L2, FABP7* and *CA9* showed the highest up-regulation. *AQP2, UMOD* and *FXYD4* were the most down-regulated genes. We generated a heat map (Figure 2A) and a volcano map (Figure 2B) to visualize these changes.

**Table 1:** The top 10 most up-regulated genes

ID	logFC	AveExpr	*t*	*P*.Value	adj.*P*.Val	*B*
NDUFA4L2	4.432022	12.06456	6.953796	1.37E-08	1.67E-05	9.54055
FABP7	3.47967	10.25692	3.302987	0.001912	0.036471	-1.48766
CA9	3.150126	9.774946	5.088065	7.29E-06	0.001112	3.67016
C7orf68	2.742933	10.45873	5.710397	9.13E-07	0.000281	5.615285
NNMT	2.711802	11.98005	4.820434	1.76E-05	0.002008	2.847483
ENPP3	2.556364	9.94591	3.937775	0.000291	0.011577	0.238448
APOC1	2.543659	10.60647	4.564635	4.04E-05	0.003398	2.072613
IGFBP3	2.488282	12.56189	5.498134	1.86E-06	0.000479	4.948096
NPTX2	2.46653	9.605073	3.146039	0.002975	0.048182	-1.88811
COL1A1	2.417302	10.92768	3.910376	0.000317	0.012153	0.160864

**Table 2:** The top 10 most down-regulated genes

ID	logFC	AveExpr	*t*	*P*.Value	adj.*P*.Val	*B*
AQP2	-5.42705	8.034263	-8.39779	1.14E-10	8.94E-07	13.99143
UMOD	-5.17712	8.004149	-7.92607	5.35E-10	2.80E-06	12.55985
FXYD4	-4.82273	7.754556	-8.64228	5.15E-11	8.09E-07	14.72297
CLCNKA	-4.55268	8.580468	-7.0949	8.55E-09	1.41E-05	9.983262
TACSTD2	-4.2747	9.495104	-7.44668	2.63E-09	6.35E-06	11.08102
ATP6V1B1	-3.98328	8.377315	-6.95776	1.36E-08	1.67E-05	9.552989
KNG1	-3.88393	7.747917	-6.78814	2.40E-08	2.39E-05	9.019412
MAL	-3.86812	9.489198	-5.66375	1.07E-06	0.000319	5.468436
ALDOB	-3.67897	8.793396	-4.88641	1.42E-05	0.001745	3.049272
CLDN8	-3.43547	7.761461	-6.123	2.27E-07	0.000107	6.918071

### Compound regulation network of BSJPF

**Figure 3:**
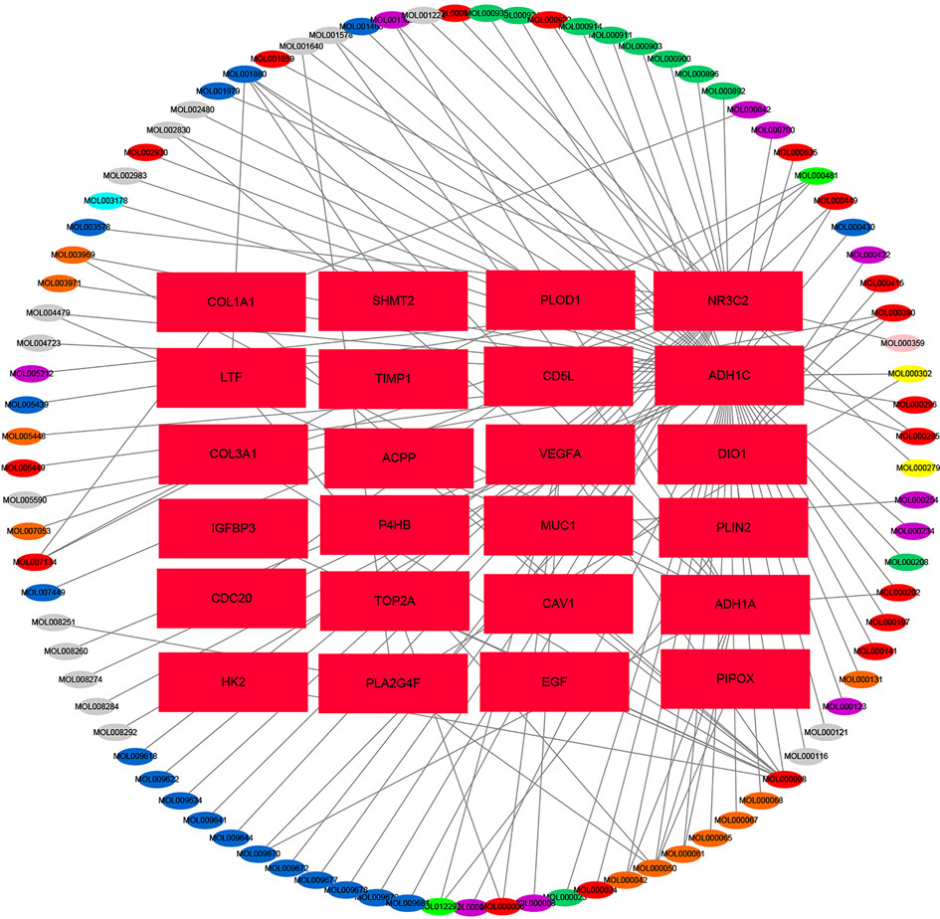
A network of BSJPF active ingredients and molecular targeting relationships There are 89 drug nodes (elliptical nodes, angelica is gray, Salvia is orange, Poria is yellow, Pueraria is grass green, wolfberry is dark blue, Coptis is sky blue, privet is purple, Curcuma is dark green, coix seed is pink, multiple drugs are combined in red) and 24 gene nodes (rectangular nodes).

A total of 922 components and 8454 targets of nine Chinese herbal medicines in BSJPF were collected through the TCMIP database. After combining them one by one, we obtained 7809 combinations. We established a decomposition network of the active ingredients in BSJPF and their molecular relationships, resulting in 113 nodes and 128 edges. We visualized the core network using Cytoscape software. There were 89 drug nodes (elliptical nodes, Angelica is shown in gray, Salvia in orange, Poria in yellow, Pueraria in grass green, wolfberry in dark blue, Coptis in sky blue, Privet in purple, Curcuma in dark green, Coix seed in pink and multiple drugs combined in red) and 24 gene nodes (rectangular nodes; Figure 3).

### Topology analysis of the PPI network

**Figure 4:**
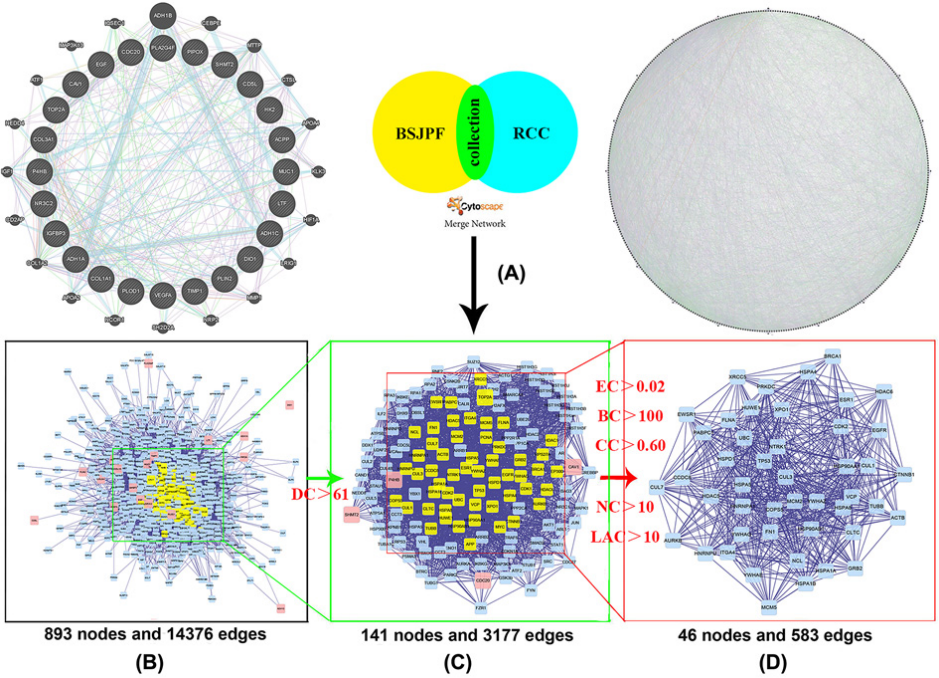
Identification of a core PPI network for BSJPF against ccRCC (**A**) Construction of two PPI networks of BSJPF targets of ccRCC. (**B**) The interactive PPI network of BSJPF and clear cell renal cell carcinoma target comprising 893 nodes and 14376 edges is shown. (**C**) PPI network of significant proteins extracted from this network comprises 141 nodes and 3177 edges. (**D**) PPI network of significant proteins extracted from (**C**); this network is made up of 46 nodes and 583 edges; BC, betweenness centrality; CC, closeness centrality; DC, degree centrality; EC, eigenvector centrality; LAC, local average connectivity; NC, network centrality.

To further understand the potential targets of BSJPF, we constructed PPIs for BSJPF targets and ccRCC differentially expressed gene targets (Figure 4A,B). To reveal the pharmacological mechanism of BSJPF in ccRCC, we combined two large networks to construct a complex network (893 nodes and 14,376 edges) (Figure 4B). Then, we conducted a topology analysis of the network to confirm the core network. Our selection criteria were as follows: BC value > 100, CC > 0.60, DC > 61, EC > 0.02, NC > 10 and LAC > 10 (Figure 4C,D). Finally, a total of 46 nodes and 583 edges were identified as the core network.

### Enrichment analysis and KEGG signaling pathway analysis of candidate targets for the mechanism of BSJPF in ccRCC

**Figure 5:**
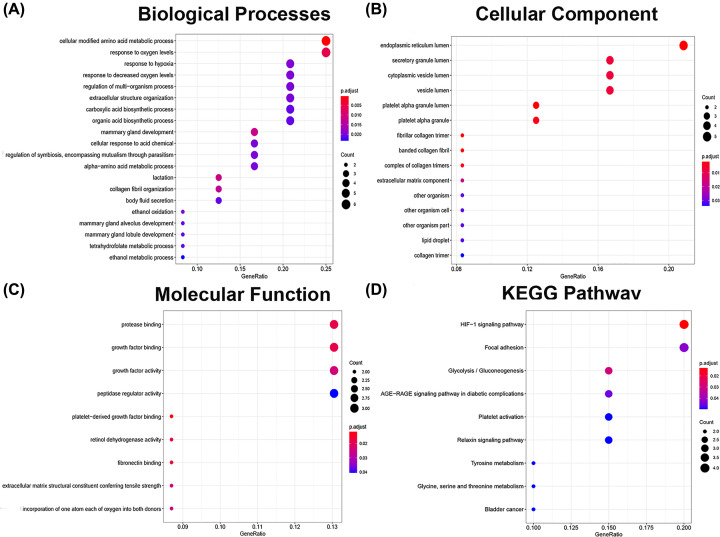
Enrichment analysis and KEGG signaling pathway analysis of candidate targets for the mechanism of BSJPF in ccRCC The top 10 terms for (**A**) biological processes, (**B**) cell component, (**C**) molecular function and (**D**) KEGG pathway.

After screening 24 candidate targets for BSJPF in ccRCC and conducting GO analysis using the DAVID database to explore the connections between functional units, we evaluated their potential importance in biological system networks, and what BSJPF passed. This method has an impact on ccRCC and the therapeutic effect. We selected three parts of the GO analysis as the main research objects: biological processes (Figure 5A), cellular components (Figure 5B), and molecular functions (Figure 5C). We found that biological processes were related to cellular modified amino acid metabolic process, response to oxygen levels, mammary gland development and others. Cellular components were related to the endoplasmic reticulum lumen, platelet alpha granule lumen and fibrillar collagen trimer. Finally, molecular functions were related to platelet-derived growth factor binding, retinol dehydrogenase activity, fibronectin binding and others. We also conducted KEGG enrichment analysis on these data and found that they were mainly related to the HIF-1 signaling pathway, glycolysis/gluconeogenesis and focal adhesions (Figure 5D).

### KEGG relationship network construction

**Figure 6:**
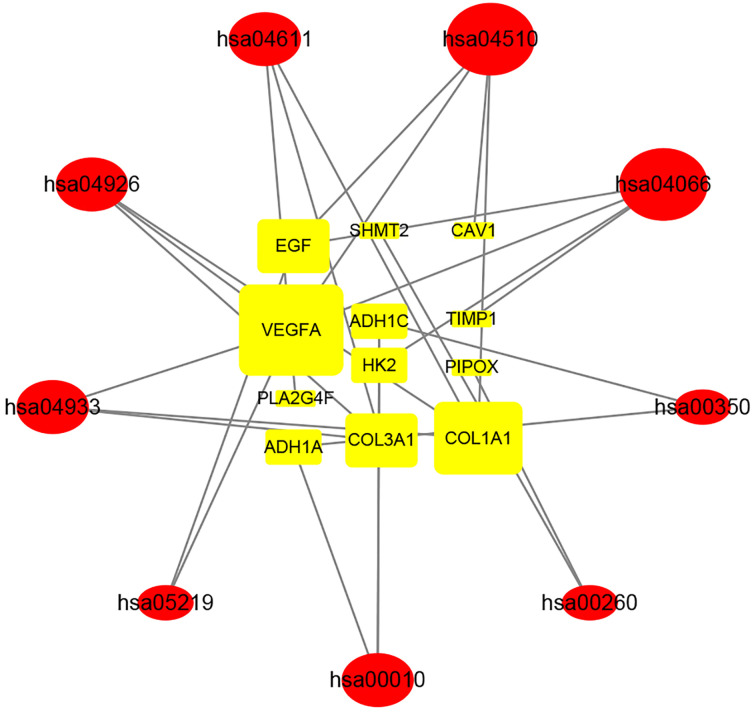
Network of compounds of BSJPF Decoction-drug targets-atherosclerosis targets-signaling KEGG pathway 12 genes (rectangular) and 9 KEGG signal pathways (oval) associated with them, the larger the number of adjacent nodes, the larger the graph.

A total of 24 significant DEGs and 9 KEGG pathways mainly related to tumor invasion and proliferation were screened and identified. *VEGFA, COL1A1, COL3A1, EGF, HK2, SHMT2, PLA2G4F, ADH1A, ADH1C, CAV1, TIMP1* and *PIPOX* were selected as drugs targets of BSJPF in ccRCC patients. As shown in Figure 6, we obtained 12 genes (rectangular) and 9 KEGG signal pathways (oval) associated with them. The greater the degree of association with adjacent nodes, the greater the shape of the nodes. We analyzed nine KEGG pathways and found that they were related to tumor proliferation, invasion and microvascular growth, indicating that BSJPF inhibits ccRCC through the above target genes.

### Prognostic value of hub genes related to BSJPF

**Figure 7:**
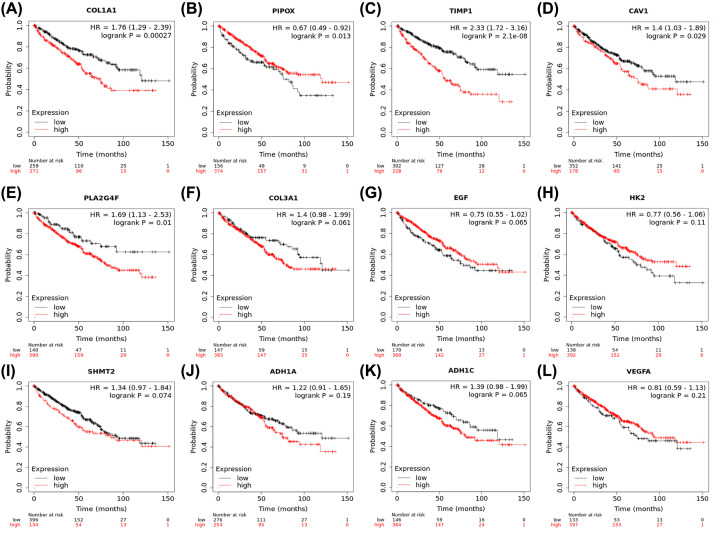
Prognostic value of hub genes related to BSJPF for ccRCC patients from TCGA cohort (**A–L**) Survival plots were performed in 12 hub genes, including *VEGFA, COL1A1, COL3A1, EGF, HK2, SHMT2, PLA2G4F, ADH1A, ADH1C, CAV1, TIMP1* and *PIPOX.* It suggested that *COL1A1, PIPOX, TIMP1, CAV1* and *PLA2G4F* were significantly associated with prognosis for 530 ccRCC patients from TCGA database (*P*<0.05). Each survival analysis was implemented, all possible cutoff values between the lower and upper quartiles are computed, and the best performing threshold is used as a best cutoff in the present study.

Survival plots were performed based on 12 hub genes, including *VEGFA, COL1A1, COL3A1, EGF, HK2, SHMT2, PLA2G4F, ADH1A, ADH1C, CAV1, TIMP1* and *PIPOX.* The results suggested that *COL1A1, PIPOX, TIMP1, CAV1* and *PLA2G4F* were significantly associated with the prognosis of 530 ccRCC patients from the TCGA database (*P*<0.05, Figure 7A–L).

### BSJPF suppresses proliferation and reshapes the tumor microenvironment in ccRCC xenograft mouse model

**Figure 8:**
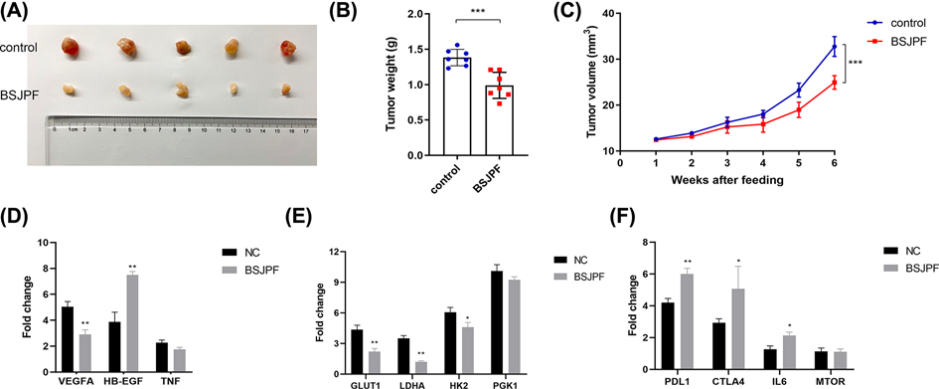
BSJPF suppresses proliferation and reshape the tumor microenvironment in ccRCC xenograft mouse model (**A–C**) To further confirm the effect of BSJPF on ccRCC cell proliferation, a xenograft mouse model was constructed by subcutaneously injecting nude mice with RENCA cells. Tumor size and volume were significantly inhibited in BSJPF group compared with normal control. In order to detect whether BSJPF affecting the predicted hub genes using bioinformatics, potential targets involved in glycolysis and tumor immune microenvironment bas been evaluated. (**D**) Expression levels of VEGF-A marked decreased and heparin binding-EGF expression was increased in BSJPF group. (**E**) Importantly, it suggested that BSJPF could inhibits tumor proliferation via enhancing GLUT1- and LDHA-related glycolysis. (**F**) Interestingly, BSJPF also significantly increase the expression of immune checkpoint molecules PDL1 and CTLA-4. It indicated that BSJPF may shape the immune-rejection type tumor microenvironment, which may allow ccRCC patients to benefit from immunotherapy.

To further confirm the effect of BSJPF on ccRCC cell proliferation, a xenograft mouse model was constructed by subcutaneously injecting nude mice with RENCA cells. When tumors reached 120 mm^3^, mice were randomized into two groups (*n*=5 each) and treated with a decoction of BSJPF or normal feeding. Tumor size and volume were significantly decreased in the BSJPF group compared with the normal control group (Figure 8A–C). To determine whether BSJPF affected the predicted hub genes using bioinformatics methods, potential targets involved in regulating glycolysis and the tumor immune microenvironment were evaluated. The expression levels of VEGF-A were markedly decreased, and heparin binding-EGF expression was increased in the BSJPF group (Figure 8D). Importantly, these results suggested that BSJPF inhibits tumor proliferation by enhancing GLUT1- and LDHA-related glycolysis (Figure 8E). Interestingly, BSJPF also significantly increased the expression of the immune checkpoint molecules PDL1 and CTLA-4 (Figure 8F). Therefore, BSJPF may alter the immune-rejection status of the tumor microenvironment, thereby allowing ccRCC patients to benefit from immunotherapy.

## Discussion

In the present study, we first screened the target genes related to ccRCC and identified the genes that affect the occurrence, development and prognosis of ccRCC. Then, the active ingredients and potential targets of BSJPF that may affect prognosis were explored using online pharmacology databases. Our research lays the foundation for further research on the treatment of ccRCC with Chinese medicine.

In the past, scientists have explored the potential targets of various drugs and verified these candidates in basic science experiments or clinical trials [24]. However, the development of big data and pharmacological methods has provided researchers with increased opportunities to analyze the relationship between drugs and diseases using bioinformatics networks [25]. Network pharmacology is a valuable tool to explore drug pathway regulation, thereby improving the efficacy of drugs and the success rate of clinical trials and reducing drug development costs [26,27]. TCMs and their active ingredients are promising prospects in the treatment of many complex diseases [28,29]. Therefore, the present study aimed to understand the biological mechanism of BSJPF in ccRCC based on network pharmacology and mouse model analyses.

Studies conducted in Asia have shown that some components of BSJPF play an important role in the treatment of renal disease. For example, BSJPF could provide nutrients and improve the circulatory system of the kidney [30]. Researchers also showed that some components of BSJPF exhibit antiapoptosis, antioxidant and anti-inflammatory activities, which are conducive to the restoration of kidney function [31–33]. In the present study, we conducted GO analysis and found that BSJPF participated in many biological processes related to tumorigenesis and development, such as cellular modified amino acid metabolic processes and fibronectin binding. By dividing the KEGG pathways of the core PPI network, we found that BSJPF may play a role in the treatment of ccRCC by regulating malignant cell behaviors. According to the *P* value of each enrichment pathway and its relationship with ccRCC, we identified nine relatively important related signaling pathways, which are closely associated with tumor proliferation and invasion.

Tumors maintain the balance of energy supply and demand through a metabolic reprogramming mode that is different from normal tissues, thereby protecting their own survival. The occurrence and development of ccRCC often involve changes in a wide range of metabolic pathways, such as glycolysis, oxidative phosphorylation and hypoxia. The proliferation, metastasis and other activities of tumor cells require glucose decomposition for energy. In the present study, potential targets of BSJPF involved in glycolysis and the tumor immune microenvironment were evaluated. The expression levels of VEGF-A were markedly decreased, and heparin binding-EGF expression was increased in the BSJPF group. Importantly, this suggested that BSJPF could inhibit tumor proliferation by enhancing GLUT1- and LDHA-related glycolysis. The HIF-1 signaling pathway is regulated by Von Hippel–Lindau tumor suppressor protein and induces glucose metabolism, cell proliferation and angiogenesis, thereby playing a significant role in ccRCC progression [34–36]. This remodeling of energy metabolism provides tumor cells with growth and proliferation advantages, helps tumor cells escape apoptosis and creates a tumor microenvironment that is more conducive to metastasis [37]. Interestingly, BSJPF also significantly increased the expression of the immune checkpoint molecules PD-L1 and CTLA-4, indicating that it may alter the immune-rejection status of the tumor microenvironment and allow ccRCC patients to benefit from immunotherapy [38]. Traditional Chinese drugs combined with immunotherapy and targeted therapy have become the mainstay of treatment for advanced renal cell carcinoma. However, more efficient treatments are likely to be identified, and traditional Chinese medicine could be one of the most promising treatment strategies. Furthermore, investigating the metabolic reprogramming of tumors and immune cells in the tumor microenvironment is critical to understanding the biological behavior of tumor cells, tumor immune responses and tumor immune escape and provides new mechanisms contributing to the regulation of tumor immunity [23,39]. Therefore, the targeted inhibition of tumor aerobic glycolysis (the Warburg effect) may bring new perspectives, strategies and evidence for the development of treatments.

There are some limitations to this research. First, most experiments were performed *in silico* and based on complex bioinformatics methods. Second, experimental sorting was not as generally expected. The present study failed to perform fundamental experiments with BSJPF and did not investigate the interactions among all potential targets. Third, the present study did not provide sufficient clinical evidence to prove the effects of BSJPF on the recurrence and long-term survival of ccRCC patients, and there is no real-world verification of the identified biomarkers.

## Conclusion

In summary, the present study demonstrated that the mechanism of BSJPF involves multiple targets and signaling pathways related to tumorigenesis and glycolysis metabolism in ccRCC. Our research provides a novel theoretical basis for the treatment of tumors with traditional Chinese medicine and new strategies for immunotherapy in ccRCC patients.

## Supplementary Material

Supplementary Table S1

## Data Availability

The datasets analyzed in this study were obtained from the corresponding author upon reasonable request or open-access online databases.

## Abbreviations

BSJPF: Bu-Shen-Jian-Pi-Fang
ccRCC: clear cell renal cell carcinoma
GO: Gene Ontology
KEGG: Kyoto Encyclopedia of Genes and Genomes
PPI: protein–protein interaction
RCC: renal cell carcinoma
TCGA: the Cancer Genome Atlas
TCM: traditional Chinese medicine
TCMSP: Traditional Chinese Medicine System Pharmacology
