# Supplementary material for: Traditional Chinese medicine Bu-Shen-Jian-Pi-Fang attenuates glycolysis and immune escape in clear cell renal cell carcinoma: results based on network pharmacology
Source: Biosci Rep. 2021 Jun 10;41(6):BSR20204421. doi: 10.1042/BSR20204421 (PMC8202066; doi:10.1042/BSR20204421)
Supplement: Supplementary Table S1 [file BSR-2020-4421_supp.pdf]

Table S1. List of primers sequencing from 5’ to 3’ used for RT-qPCR

Genes symbols	Primers sequencing
VEGFA	F - AGG GCA GAA TCA TCA CGA AGT<br>R - AGG GTC TCG ATT GGA TGG CA
HB-EGF	F – ATC GTG GGG CTT CTC ATG TTT<br>R – TTA GTC ATG CCC AAC TTC ACT TT
TNF	F - GAG GCC AAG CCC TGG TAT G<br>R - CGG GCC GAT TGA TCT CAG C
GLUT1	F - TAG AAA CAT GGT TTT GAA ATG CTT<br>R - CCC ACT TAC TTC TGT CTC ACT CC
LDHA	F - TGG AAA TAT TAG GCT ATT CTT GGG C<br>R - GAC TAT TAC ATC CTC TGC TAT TAG TC
HK2	F – ATC CCT GAG GAC ATC ATG CGA<br>R – CTT ATC CAT GAA GTT AGC CAG GCA
PGK1	F - CCG CTT TCA TGT GGA GGA AGA AG<br>R - CTC TGT GAG CAG TGC CAA AAG C
PDL1	F - TGC CGA CTA CAA GCG AAT TAC TG<br>R - CTG CTT GTC CAG ATG ACT TCG G
CTLA4	F - CCA GTT CCT TTC AAT GGT TTG C<br>R - GCC CAT CGA ACT GGA GC
IL6	F - GGC ACT GGC AGA AAA CAA CC<br>R - GCA AGT CTC CTC ATT GAA TCC
MTOR	F - AGC ATC GGA TGC TTA GGA GTG G<br>R - CAG CCA GTC ATC TTT GGA GAC C
β-actin	F –CCT CGC CTT TGC CGA TC<br>R- GGA TCT TCA TGA GGT AGT CAG TC
